# Mass Spectrometry-Based Comprehensive Analysis of Pancreatic Cyst Fluids

**DOI:** 10.1155/2018/7169595

**Published:** 2018-11-29

**Authors:** Agnieszka Paziewska, Marcin Polkowski, Tymon Rubel, Jakub Karczmarski, Anna Wiechowska-Kozlowska, Michalina Dabrowska, Michal Mikula, Michal Dadlez, Jerzy Ostrowski

**Affiliations:** ^1^Department of Gastroenterology, Hepatology and Clinical Oncology, Medical Center for Postgraduate Education, Warsaw, Poland; ^2^Institute of Radioelectronics and Multimedia Technology, Warsaw University of Technology, Warsaw, Poland; ^3^Department of Genetics, Maria Sklodowska-Curie Memorial Cancer Center and Institute of Oncology, Warsaw, Poland; ^4^Department of Endoscopy, Ministry of Internal Affairs Hospital, Szczecin, Poland; ^5^Institute of Biochemistry and Biophysics, Polish Academy of Sciences, Warsaw, Poland

## Abstract

Pancreatic cyst fluids (PCFs) enriched in tumour-derived proteins are considered a potential source of new biomarkers. This study aimed to determine compositional and quantitative differences between the degradome and proteome of PCFs aspirated from different types of pancreatic cyst lesions (PCLs). 91 patients who underwent endoscopic ultrasound-fine needle aspiration under routine clinical diagnosis of PCLs were enrolled. Four cysts were malignant (CAs), and 87 were nonmalignant and consisted of 18 intraductal papillary mucinous neoplasms (IPMNs), 14 mucinous cystic neoplasms (MCNs), nine serous cystic neoplasms (SCNs), 29 pseudocysts (PCs), and 17 unclassified. Profiles of the <5 kDa fraction, the degradome, and the trypsin-digested proteome were analysed using an LTQ-Orbitrap Elite mass spectrometer coupled with a nanoACQUITY LC system. Qualitative analyses identified 796 and 366 proteins in degradome and proteome, respectively, and 689 (77%) and 285 (78%) of them were present in the Plasma Proteome Database. Gene Ontology analysis showed a significant overrepresentation of peptidases and peptidases inhibitors in both datasets. In the degradome fraction, quantitative values were obtained for 6996 peptides originating from 657 proteins. Of these, 2287 peptides were unique to a single type, and 515 peptides, derived from 126 proteins, were shared across cyst types. 32 peptides originating from 12 proteins had differential (adjusted* p*-value ≤0.05, FC ≥1.5) abundance in at least one of the five cysts types. In proteome, relative expression was measured for 330 proteins. Of them, 33 proteins had significantly (adjusted* p*-value ≤0.05, FC ≥1.5) altered abundance in at least one of the studied groups and 19 proteins appeared to be unique to a given cyst type. PCFs are dominated by blood proteins and proteolytic enzymes. Although differences in PCF peptide composition and abundance could aid classification of PCLs, the unpredictable inherent PCF proteolytic activity may limit the practical applications of PCF protein profiling.

## 1. Introduction

The prevalence of pancreatic cysts in different studied populations varies between 2% and 45% [1]. Clinically, they are categorised as nonneoplastic and neoplastic. The most common pancreatic nonneoplastic cysts are pseudocysts (PCs), while neoplastic cysts are represented by serous cystic neoplasms (SCNs), mucinous cystic neoplasms (MCNs), and intraductal papillary mucinous neoplasms (IPMNs) [1]. To recognise the cyst type, clinical presentation, and patient's age and sex, axial imaging based on computed tomography (CT) or magnetic resonance imaging (MRI) and endoscopic ultrasound-fine needle aspiration (EUS-FNA) results are combined in clinical practice [2]. However, the diagnostic accuracy of CT- and MRI-based imaging techniques ranges from 55% to 75% [1], while the sensitivity of cytological evaluation of cyst fluid is ~50% for differentiation of pancreatic cyst lesions (PCLs) [3–5].

Advances in proteomic methodology have stimulated substantial interest for investigating PCF proteomes (reviewed in [6]), and a few potential candidate biomarkers have been reported, including olfactomedin-4, several mucins, two homologs of amylase, four solubilised carcinoembryonic antigen (CEA)-related cell adhesion molecules, four S100 homologs, and IL1*β* [2, 7, 8]. In addition, glycan variants on mucin proteins appear to be particularly sensitive and specific for differentiating between mucinous cysts [9, 10], while high-mobility group (HMG) A2 protein is considered a dysplasia grade biomarker in IPMNs [11]. However, except for CEA and amylase, no other protein marker by itself is consistently reliable and sufficiently conclusive for diagnosing and risk-stratifying pancreatic cysts.

The biological nature of pancreatic cysts varies significantly because their fluids can be dense or diffuse, mucinous, or bloody, mixed together with the contents of pancreatic ducts or isolated from pancreatic proenzymes [8]. Based on type, pancreatic cyst fluids (PCFs) are divided into serous (containing a thin fluid) or mucinous (containing a viscous fluid). However, in contrast to the relatively constant composition of serum proteins, differences within proteomes from the same type of PCFs are apparent, making PCF proteomics challenging. One of the fundamental causes of these differences is the variable abundance and combination of pancreatic peptidases, including carboxypeptidases, aminopeptidases, and matrix metalloproteases. The proteolytic activity of these enzymes results in a constellation of peptides derived from major proteins (the degradome), and studying these peptides (degradomics) is likely to prove useful for PCF research.

To date, no single study has compared the degradome and proteome of PCFs. Herein, we present a qualitative and quantitative MS-based survey of the degradome and proteome of nonprotease inhibitors treated PCFs derived from clinically different cyst types.

## 2. Materials and Methods

### 2.1. Study Participants

The study protocol was accepted by the Ethical Review Board at the M. Skłodowska-Curie Memorial Cancer Centre and Institute of Oncology, Warsaw, Poland. The study was conducted according to the principles expressed in the Declaration of Helsinki and informed written consent was obtained from the participants. From March 2012 to November 2013, 91 patients were recruited at two large-volume EUS-centers: Department of Gastroenterology, M. Skłodowska-Curie Memorial Cancer Centre, Warsaw, Poland, and Department of Endoscopy, Ministry of Internal Affairs Hospital, Szczecin, Poland. Cyst fluids were collected prospectively and the analyses were performed as a part of routine diagnostic work-up. Inclusion criteria were as follows: (1) Patients aged 18 years or older. (2) A cyst larger than 15 mm. (3) Written patient's informed consent for participating in the study. Exclusion criteria were as follows: (1). Known coagulation disorder (INR>1,5, PTT>50 seconds, platelets<50.000/*μ*L). (2) Being treated with acetylsalicylic acid and/or clopidogrel within 5 days.

EUS-FNA was performed by experienced endosonographers (MP, AWK) in standard fashion with a curved-linear echoendoscope and 22G or 19G needles. The following PCL characteristics were recorded: cyst location, maximal diameter, and morphology: wall thickness, septations, calcifications, solid component, and mural nodules. An attempt at total aspiration of the cyst fluid was made whenever possible. Cyst fluids were analysed by standard diagnostic procedures (cytology, amylase, CEA, and viscosity), and rest of the aspirated fluids were aliquoted into Eppendorf tubes at 1ml volumes and stored at −80°C within 30 min of harvesting. The protease inhibitors were not spiked-in to PCF upon collection. Targeted cyst wall punctures were performed at the endosonographer discretion. Prophylaxis with fluoroquinolones or betalaktam antibiotics was administered according to the European Society of Gastrointestinal Endoscopy guideline [13]. Patients were observed for at least two hours postprocedure.

### 2.2. Preparation of Cysts Fluid Samples for Peptidome Analyses

The LMW fraction of the PCFs was analysed as described previously for the plasma/serum peptidome [14]. Briefly, a mix of 100 *μ*l PCF + 300 *μ*l 25% acetonitrile in 25 mM NH_4_HCO_3_ was centrifuged at 5000 x g, at 15°C, for 90 minutes through a 30 kDa cutoff filtration membrane (Millipore Ultrafree-MC, pore size 30000, Sigma-Aldrich), and then washed twice with 25% acetonitrile prior to use. Next, 100 *μ*l 25% acetonitrile was applied to the filter and again centrifuged for 60 min. Finally, the combined filtrates were centrifuged at 5000 x g, at 15°C, for 120 minutes through the 5 kDa cutoff filtration membrane (Millipore Ultrafree-MC, pore size 5000), and the <5 kDa fraction was lyophilized (Speed Vac) in siliconized microtubes (Clear-view Snap-Cap microtubes, size 1.5 mL, low retention, Sigma-Aldrich) and stored at –80°C for further use. Prior to LC-MS analysis, samples were dissolved in 0.1% trifluoroacetic acid (TFA). MS/MS data was acquired from pooled PCF samples prepared by combining 10 *μ*l of fluids belonging to given cyst type from the study samples.

### 2.3. Preparation of Cysts Fluid Samples for Proteome Analyses

Proteins were precipitated from equal amounts (by protein content) of PCF samples using the ProteoExtract® Protein Precipitation Kit (Calbiochem Cat. No. 539790) according to the manufacturer's protocol. Protein pellets were resuspended in 100 *μ*l 100mM NH_4_HCO_3_, the protein concentrations were then measured by the BCA method (Pierce™ BCA Protein Assay Kit) and aliquots equal to 20 *μ*g proteins were reduced in 5mM Tris-(2-carboxyethyl) phosphine (Sigma-Aldrich), alkylated with 8mM S-Methyl methanethiosulfonate (Sigma-Aldrich), then trypsin-digested (Sequencing Grade Modified Trypsin, Promega) using standard protocols, and finally acidified with 5%TFA to pH=4.

### 2.4. LC-MS Settings

LC-MS analysis was performed on a LTQ-Orbitrap Elite mass spectrometer (Thermo) coupled to a nanoAcquity (Waters) LC system as described before [15]. Qualitative LC-MS/MS analyses were carried out on pooled samples in data-dependent acquisition mode and peptide fragmentation was achieved by high-energy collision dissociation (HCD). To increase the number of peptide identifications, three LC-MS/MS runs were performed per pooled sample, each covering one of three ranges of* m/z *values: 300–600, 600–800, and 800–2000. Quantitative analyses of individual samples were performed using separate survey scan LC-MS runs with a m/z measurement range of 300–2 000 and the same acetonitrile gradient settings as those used for the LC-MS/MS runs.

### 2.5. Qualitative MS Data Processing and Database Search

The MS/MS raw data files were submitted to Mascot Distiller (version 2.5.1, Matrix Science), and the subsequent peak lists were uploaded to the Mascot engine (version 2.4.1, Matrix Science) and searched against SwissProt Homo sapiens database (release 2016.01) supplemented with contaminant proteins sequences from the Repository of Adventitious Proteins (cRAP, http://www.thegpm.org/crap). In total, the database contained 20,233 target sequences, and the same number of reversed decoy records. The search parameters were as follows: enzyme specificity: semitrypsin for proteome samples, and none for the degradome; maximum number of missed cleavages (for proteome only): 1; protein mass: unrestricted; parent ions mass error tolerance: 5 ppm; fragment ions mass error tolerance: 0.01 Da; fixed modifications (for proteome only): Methylthio (C); variable modifications: Oxidation (M).

Statistical significance of identified peptides was calculated as previously described [16, 17]. For proteome samples proteins represented by less than two peptides or identified by peptides matching other proteins were excluded from analysis. Proteins matching the same set of peptides were merged together into metaproteins clusters.

As a graphical representation of protease specificity in degradome samples Sequence Logo was used [18]. Peptides identified in the studied group of samples were aligned to corresponding proteins, and amino acids at positions flanking the location their N- and C-termini were recorded. Next sequence logos depicting relative frequencies of amino acids around cleavage sites were generated.

Mascot results processing was done using MScan software available at http://proteom.ibb.waw.pl/mscan. This software tool was also used for Sequence Logo generation.

### 2.6. Quantitative MS Data Processing

The quantitative MS data processing and peptides feature extraction procedure was described in detail in a previous study [9]. Log-transformed peptide abundances were normalized by fitting a robust locally weighted regression smoother (LOESS) between individual samples and a median pseudosample. LOESS is a well-established method of normalization, commonly used to minimize systematic bias and the effects of nonbiological sources of variation in microarray and proteomic datasets [19, 20]. The parameters for the fit were established using a set of features that exhibited low variance in the nonnormalized data and then applied to the whole data set. In the case of proteome samples, the normalized peptide-level data were rolled-up to relative protein abundances. The procedure used involved rescaling abundances of peptides that originated from the same protein to a common level, followed by computing their median value.

### 2.7. Statistical Analysis

For peptide and protein abundance comparisons a nonparametric resampling-based ANOVA (Analysis of Variance) test with the F-statistic was used. The resulting* p*-values were corrected for multiple hypothesis testing by the Benjamini–Hochberg procedure for controlling the false discovery rate (FDR) [21]. Only peptide or protein abundances with FDR-adjusted* p*-values ≤ 0.05 and fold change (FC) values ≥ 1.5 were considered significantly changed.

Unsupervised principal components analysis (PCA) was utilized to evaluate and visualize the relationships between cyst types. PCA was computed using singular values decomposition (SVD) of both quantitative datasets.

All statistical analyses were performed in MStat (available at http://proteom.ibb.waw.pl/) running in the MATLAB (MathWorks) environment.

### 2.8. Functional Analysis

Identified proteins were annotated with Gene Ontology (GO) terms using the Molecular Function GO-Slim from the PANTHER web service (version 13.1) [22]. The binomial test, implemented in PANTHER, was used to determine the statistical significance of under- or overrepresentation of GO terms, and the returned* p*-values were corrected for testing of multiple hypotheses with the Bonferroni method. Adjusted* p*-values less than or equal to 0.05 were considered significant.

## 3. Results

### 3.1. Patient and Cyst Characteristics

**Figure 1:**
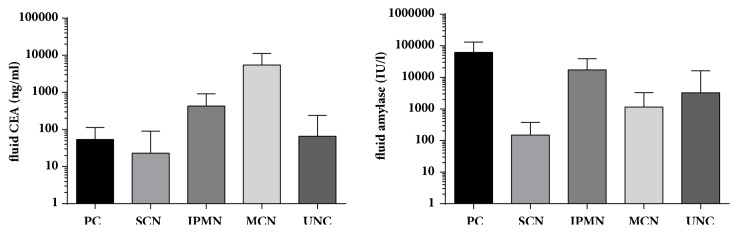
Comparison of cyst fluid carcinoembryonic antigen (CEA) and amylase levels in patients with pancreatic lesions (results presented on a logarithmic scale). Whiskers denote standard deviation. PC, pseudocyst; SCN, serous cystic neoplasm; IPMN, intraductal papillary mucinous neoplasm; MCN, mucinous cystic neoplasm; UNC, unclassified.

The cohort consisted of 91 patients who underwent EUS-FNA under routine clinical diagnosis of PCLs. Clinical features of patients, including general demographics, cyst characteristics, and the cyst fluid concentration of CEA and amylase, are summarised in Table 1. Based on surgical pathology or, in patients in whom PCLs were not resected, on clinical follow-up for at least 1 year, four PCLs were diagnosed as malignant (one invasive adenocarcinoma with cystic degeneration, two MCNs and one IPMN with high-grade dysplasia). The remaining 87 PCLs were nonmalignant, classified as mucinous (18 IPMNs and 14 MCNs) and nonmucinous (29 PCs and 9 SCNs), while 17 PCLs were unclassified (UNC). Sufficient cyst fluid was allocated for cytology and molecular analyses for all 91 patients, and fluid CEA and amylase were measured in 86 (94.5%) PCLs. Cyst fluid concentrations of CEA and amylase are summarised in Figure 1.

Mucinous cysts are lined with endoderm-derived columnar epithelium that secretes CEA, whereas nonmucinous cysts are lined by simple nonendoderm-derived epithelium that produces little or no CEA [23]. CEA abundance is the single best indicator for discriminating the malignant potential of PCLs, which, depending on the established cutoff level, may reach a diagnostic accuracy of 80%; hence it is considered the discriminatory method of choice for mucinous and nonmucinous PCLs [6, 7, 24, 25]. We confirmed that at a cutoff value of 192 ng/mL, PCF CEA was moderately sensitive and highly specific for diagnosing all mucinous cysts (sensitivity = 81.2%; specificity = 91.6%). The diagnostic accuracy of the test was 86.7%, and the area under the curve (AUC) was 0.93.

High levels of cystic fluid amylase have been found in pancreatic cysts that communicate with pancreatic ducts [26]. Pancreatic PCs usually communicate with pancreatic ducts, while IPMNs occupy part or the whole length of the main pancreatic duct and/or side pancreatic branches, and fluids from both cyst types are rich in amylase and other pancreatic enzymes. The cavities of both MCNs and SCNs rarely communicate with pancreatic ducts [24]. Unsurprisingly, the sensitivity of an amylase cutoff value of 250 UI/L [27] for combined PCs and IPMNs was high (97.7%), and the specificity (62.5%), accuracy (85.3%), and AUC (0.945) were also relatively high.

### 3.2. Peptidome Analysis

LC-MS/MS analyses of the <5 kDa fraction from all cyst types were performed to establish a MS/MS database of the low molecular weight (LMW) peptidome fraction for further quantitative comparisons between samples. Two measurements of pooled samples were carried out for each group. The analyses yielded 411,763 fragmentation spectra, and a search against the SwissProt database using the Mascot engine identified a set of 9429 peptides (represented by 78,013 spectra), with an estimated false discovery rate (FDR) of 0.01 (Table S1). Peptides originated from 796 proteins, among which 442 were represented by at least two peptides (Table S2). Of note, 77% of proteins were present in the Plasma Proteome Database (PPD; www.plasmaproteomedatabase.org) [28], and more than 25% of identified peptides represented three blood proteins (haemoglobin, albumin, and fibrinogen) indicating extensive similarity in cyst fluid composition with the blood proteome.

Gene Ontology (GO) analysis showed a significant overrepresentation of peptidases and their inhibitors in our dataset (adj.* p* ≤ 0.05 with Bonferroni correction), assigning them into five GO terms based on the molecular function category (Figure S2 and Table S3). Overall, of the 796 identified proteins there were 60 proteins belonging to the peptidase activity GO term (Table S4), and 111 were present in the MEROPS database [29] that provides information on peptidases and their inhibitors (Table S2). Although cyst fluid proteomes are very similar to the blood proteome, it is not clear whether cyst fluid peptides derived from serum proteins reflect proteolytic activity of blood, cyst fluids or both.

**Figure 2:**
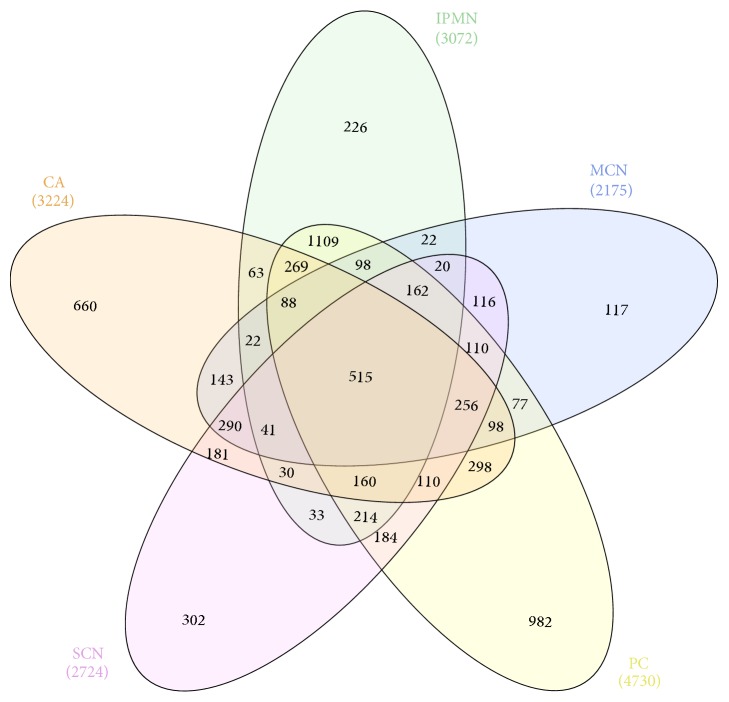
Venn diagram depicting the distribution of identified peptides in different types of pancreatic cyst lesions CA, carcinoma; SCN, serous cystic neoplasm; MCN, mucinous cystic neoplasm; IPMN, intraductal papillary mucinous neoplasm; PC, pseudocyst.

Peptides identified in all LC-MS/MS runs were then overlaid on the LC-MS profile data of individual samples to extract peptides' quantitative features across 8, 16, 5, 7, and 3 IPMN, PC, MCN, SCN, and CA PCL samples, respectively. Additionally, nine UNC samples were also included in the analysis. Quantitative values were obtained for 6996 peptides originating from 657 proteins (Table S5). Of these, 6481 peptides were not found in at least one cyst type, 2287 peptides were unique to a single type, and only 515 peptides, derived from 126 proteins, were shared across all the studied cyst types. An ANOVA-based comparison identified 32 peptides originating from 12 proteins with differential (adjusted* p*-value ≤0.05, FC ≥1.5) abundance in at least one of the five types of PCLs (Table S6). While quantitative analysis of individual degradomes has been challenging because of their noticeable variability (Table 2 and Figure 2), at least some of the observed differences might result from uneven numbers of samples representing different cyst types.

**Figure 3:**
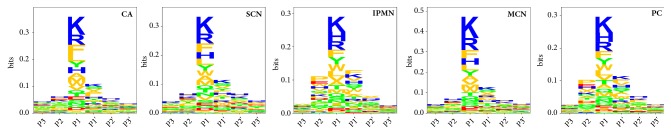
Sequence Logo plots depicting protease specificity. Positions flanking the N- and C-terminal sides of the cleavage site are named according to standard protease nomenclature, where the cleavage takes place between P1 and P1'[12]. The information content of the position is expressed in bits. Amino acids are represented in one-letter code and coloured according to their physicochemical characteristics: polar, green, hydrophobic, orange, basic, blue, acidic, red. CA, carcinoma; SCN, serous cystic neoplasm; MCN, mucinous cystic neoplasm; IPMN, intraductal papillary mucinous neoplasm; PC, pseudocyst.

**Figure 4:**
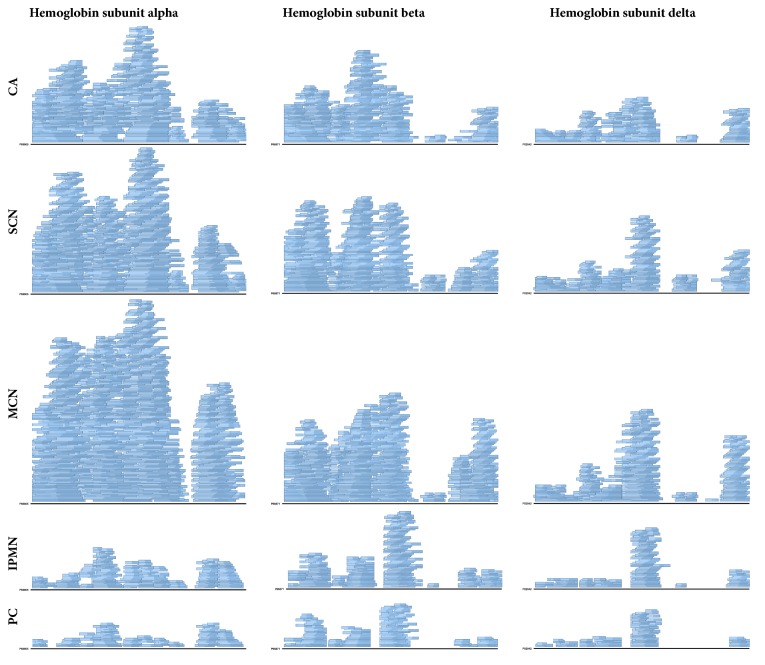
Coverage of alpha, beta and delta haemoglobin amino acid sequences in pancreatic cyst lesions. Stacked rectangles represent the distribution of identified peptides along the protein sequence. CA, carcinoma; SCN, serous cystic neoplasm; MCN, mucinous cystic neoplasm; IPMN, intraductal papillary mucinous neoplasm; PC, pseudocyst.

The comparison of sequence logos depicting relative frequencies of amino acids occurring in proteins at positions surrounding the N- and C-termini of the detected peptides revealed similarities for CA, SCN, and MCN groups, as well as for PC and IPMN samples, suggesting that differences in degradome composition may not only stem from primary protein abundance, but also from distinct proteolytic activity, inherent to a given cyst type (Figure 3). The peptide degradation patterns in CA, SCN, and MCN samples contained abundant shorter versions of initial peptide forms of a peptide ladder, as exemplified by alpha, beta and delta haemoglobin (Figure 4) and four other proteins (Figure S1), which comprised the top seven most abundant proteins in the degradome (Table S2).

**Figure 5:**
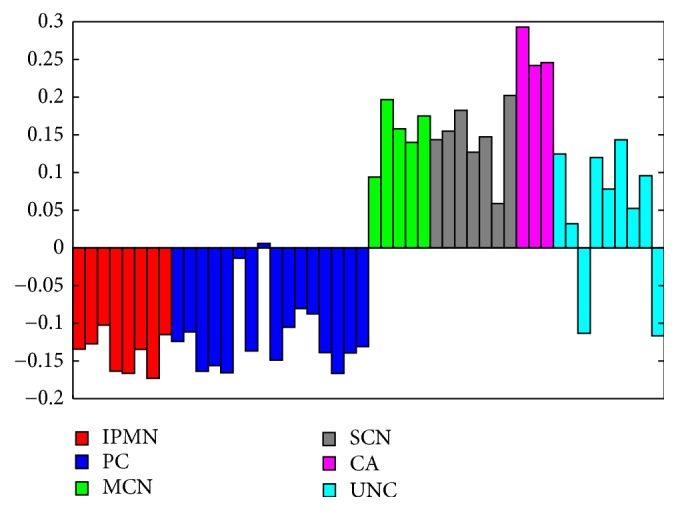
Diagram of the projection of the degradome dataset on the first principal component. IPMN, intraductal papillary mucinous neoplasm; PC, pseudocyst; MCN, mucinous cystic neoplasm; SCN, serous cystic neoplasm; CA, carcinoma.

This observation is consistent with the results of principal component analysis (PCA) of the data set. The scores of the first principal component, which captured 36% of the data variance, clearly distinguished PC and IPMN groups from CA, SCN, and MCN groups (Figure 5). Inclusion of UNC samples to the PCA analysis allowed them to be assigned to one of the supergroups. Interestingly, the differences between PC/IPMN and CA/SCN/MCN supergroups were also detectable after peptide grouping in accordance with the proteins from which they were derived. Noticeable differences in the number of peptides detected between these groups could be observed for 51 proteins (Table S7). Significantly, among these proteins the peptidases and peptidase inhibitors were present (Table 3).

### 3.3. Proteome Analysis

To catalogue cyst fluid proteomes for LC-MS/MS survey, we used trypsin-digested aspirated samples without prior fractionation. In total 393,637 MS/MS spectra were acquired. After the database searching procedure all peptides that were also present in the <5 kDa dataset were excluded from further analyses. Overall, 4011 peptides (represented by 43,370 spectra) were identified and assigned to 366 proteins with at least two peptides (Tables S8 and S9). Of these, 285 proteins (78%) were present in the PPD. Comparison of the identifications made in proteome and degradome samples revealed 239 common proteins identified by minimum two peptides, 203 unique to the <5 kDa fraction, and 127 found only in the proteome (Tables S2 and S9).

Of the identified proteins, 69 were present in the MEROPS database, and of these, 9 were not detected in the degradome dataset (Table S9). GO analysis of the proteome dataset revealed significant enrichment of 13 molecular function GO terms (Table S10 and Figure S3), of which five were related to the activity of proteases and their inhibitors. In total, 46 proteins were assigned to these GO terms, and 38 were also included in the MEROPS database (Table S11).

**Figure 6:**
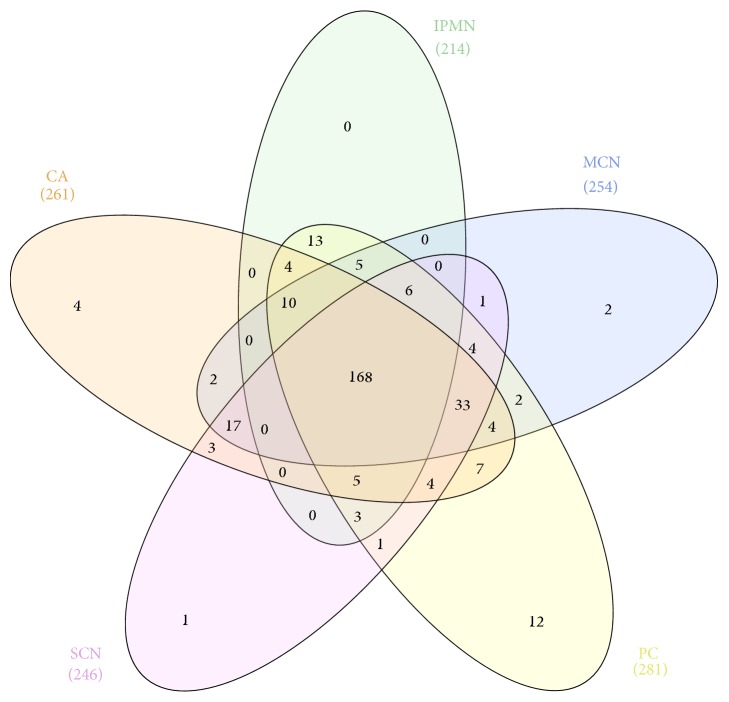
Venn diagram depicting proteome similarities in different types of pancreatic cyst lesions.

Proteome quantitative analysis of LC-MS data was performed for the same set of 48 fluid samples as employed in the degradome quantitative survey. Relative expression values in at least one individual sample were measured for 330 proteins (Table S12). Of these, 271, 246, 254, 214, and 290 proteins were found in CA, SCN, MCN, IPMN, and PC sample, respectively. As previously noted for the degradome, high compositional variability was also observed in the proteome data. For example, only about 2% of these proteins were detected in all individual samples quantified, and only 48% of proteins were present in at least 38 samples (80% of the dataset). Similarly, as in the degradome dataset, there were noticeable differences in sample composition, with only 168 (49%) common proteins, and 19 proteins which appeared to be unique to a particular cyst type (Table 4 and Figure 6). Furthermore 143 proteins were not detected in at least one of the studied groups of samples (Table S12).

**Figure 7:**
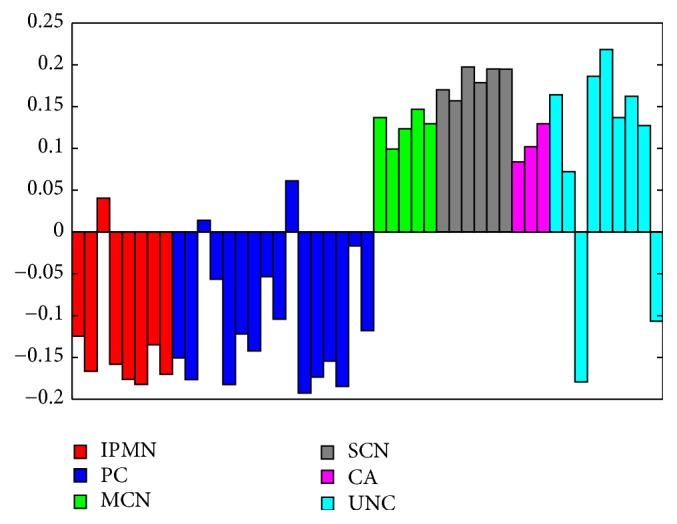
Diagram of the projection of the proteome dataset on the first principal component. IPMN, intraductal papillary mucinous neoplasm; PC, pseudocyst; MCN, mucinous cystic neoplasm; SCN, serous cystic neoplasm; CA, carcinoma; UNC, unclassified.

As before, the projection of the proteome dataset on the first principal component (representing 23% of total variance) distinguished PCs and IPMNs from CAs, SCNs, and MCNs (Figure 7). Additionally, allocation of UNC samples was consistent for both datasets (Figures 5 and 7). A further statistical testing procedure allowed for the selection of 33 proteins with significantly (adjusted* p*-value ≤0.05, FC ≥1.5) altered abundance in at least one of the studied groups (Table S13). Interestingly, 16 of the selected proteins possessed proteolytic activity according to the MEROPS database (Table S13).

## 4. Discussion

Although the concept of the peptidome and degradome has been used interchangeably, the degradome refers to the LMW fraction of the plasma/serum proteome dominated by the same endogenous peptides but shortened by one or more amino acids at the N- and/or C-termini [14, 30, 31]. The composition of the blood degradome may vary between normal and diseased phenotypes as a result of the activity of more than 500 proteases responsible for protein degradation [30]. Similarly, the presence or absence of a set of peptides in PCF, and variation in their abundance, can indicate ongoing pathologies and thus serve as potential biomarkers for the diagnosis or prognosis of pancreatic cysts.

The present study confirmed the previously published proteomic analyses of PCFs by Ke and coworkers [8] by showing that PCFs are dominated by blood proteins (80% of all identified proteins are constituents of the blood proteome), and a high proportion of identified proteins possess proteolytic activity. As a consequence, the LMW fraction of PCFs is mostly composed of proteolytic fragments of blood proteins. Contrary to the previous statement that complexity of the PCF degradome due to the presence of multiple proteases makes analysis overly challenging the differences in peptide abundance allowed us to discriminate two groups of PCFs, one comprising CA, SCN and MCN, and the other containing PC and IPMN cysts. Importantly, similarly to quantitative proteome experiments, measuring peptide levels in the degradome enabled classification of previously undiagnosed cysts into one of the groups. This was presumably possible due to the large number of different cyst types included in this study, compared with the number included in the study by Ke and colleagues who investigated PCFs from only 20 patients.

It should be noted that differences in the degradome composition are not directly transferable to differences in a given protein presence and its relative levels. As shown in Table 2, 2287 peptides unique for one of the cyst groups, which represented 35% of peptides subjected to quantitative analyses, derived from 496 proteins, represented 75% of identified proteins. Whereas peptide patterns from the given protein may be unique for a cyst type as a result of differences in cystic fluid proteolytic activity, at least to some extent, the protein itself may not be longer unique and can occur in various cyst types (Table S5). In fact, only 12, 2, 1, 4, and none proteins were unique for PC, MCN, SCN, CA, and IPMN PCFs, respectively (Table 4), and can be considered as protein markers for a given cyst type. Interestingly, some of these proteins have already been connected with pancreas biology and even proposed as potential cancer biomarkers. For example Annexin A2 (ANXA2), found in CA samples only, has been shown as aberrantly expressed in a wide spectrum of tumours, functionally playing an important role in tumour growth and progression [32]. In pancreatic cancer patient's high stromal ANXA2 level was predictive for reduced disease-free survival and overall survival [33]. The Gastricsin, in proteome dataset unique for MCN samples, has been shown as overexpressed in PCLs [34] and measurement of its activity was recently proposed as a biomarker for differentiating mucinous from nonmucinous PCLs [35]. In the PC samples we found 12 specific proteins, of them Alpha-actinin-4 (ACTN4), Keratin 18, Myosin-9, and Tropomyosin alpha-4 chain are constituents of cells' cytoskeleton. The ACTN4 expression was found in Langerhans islets at the mRNA and protein level [36]; another study reported this protein as being secreted by pancreatic cancer stem cells [37].

This is the first study comparing degradome and proteome from different types of PCFs. Although degradome-centred studies of bodily fluids are under-represented in the scientific literature compared with proteome-focused studies, exploration of endogenous, native peptides under pathogenic conditions may identify specific peptides and/or differences in their abundance that could serve as biomarkers for the diagnosis or prognosis of the disease [38]. However, the uncontrollable proteolytic activity in PCFs leads to serious methodological limitations for the use of PCF protein-based tests in clinical practice. The dominance of blood proteins in PCF samples combined with the presence of peptidases can hinder the correct identification and quantification of proteins and the effective classification of potential biomarkers when commonly used statistical methods such as unsupervised hierarchical clustering and principal component analysis are employed [39, 40]. In addition, preanalytical factors including sample collection and processing may influence PCF protein levels and composition. Indeed, in amylase-rich cyst fluids, we found noticeably fewer unique peptides and proteins, and in general, the higher the proteolytic activity in PCF, the lower the levels of intact proteins. Since we did not directly measure the enzymatic activity in cyst fluids, we can only speculate that the observed differences within the peptidome and degradome of different cyst types not only stem from variation in primary protein abundance, but may also reflect inherent and* ex vivo* proteolytic activity within a given cyst type. Unfortunately, it is not yet possible to conclude whether the cyst fluid degradome reflects proteolytic activity of pancreatic enzymes, blood proteases, or both. Additionally, future comparative experiments with the inclusion of protease inhibitors upon PCF harvesting are warranted to dissect the extent of intrinsic and* ex vivo* proteolytic activity impact on PCF proteome and degradome composition.

## 5. Conclusions

While* ex vivo* proteolytic activity can be controlled by the inclusion of protease inhibitors during sample collection,* in vivo* protein nicking in cyst fluids may not be preventable [8]. Nevertheless, we believe that peptidase-driven peptide degradation patterns could be employed for yet unexplored diagnostic purposes, opening a new avenue for investigating PCFs. Despite improvements, biomarker validation remains a challenging and time- and resource-consuming task, requiring careful consideration to reduce the number of false positive identifications during the exploratory stages of research employing mass spectrometric methods. Since no consensus has yet been developed on appropriate methods for handling and processing PCFs, similar to the difficulties encountered in the Human Plasma Proteome Project [41, 42], establishing new endogenous peptide-based biomarkers will need careful verification by alternative methods conducted on a large number of samples.

## Figures and Tables

**Table 1:** Clinical characteristics of patients.

Female/Male	Cyst type (presumptive clinical diagnosis)		Age at EUS-FNA	Max. cyst diameter (mm)	Fluid CEA (ng/ml)	Fluid amylase (IU/l)
14/15	PC	mean	56	47	54	61900
		SD	13.5	29.2	59.6	69537.8
		median	57	38	27.53	40000
		min	25	20	0.3	381
		max	81	150	211.17	301180

7/2	SCN	mean	56	39	23	149
		SD	18.2	13.2	67.0	225.9
		median	64	35	0.54	28
		min	21	24	0	10
		max	74	60	202	704

13/5	IPMN	mean	60	39	429	17338
		SD	11.4	24.4	482.8	21582.0
		median	63	35	236	2891
		min	37	15	19.31	182
		max	79	130	1435	62460

12/2	MCN	mean	62	45	5466	1154
		SD	12.5	21.8	5700.7	2122.5
		median	65.5	41.5	2931	130
		min	35	20	217.3	1
		max	74	100	18623	7106

13/4	UNC	mean	56	38	66	3266
		SD	13.4	17.6	174.3	12789.7
		median	60	38	4.03	117
		min	34	20	0.2	1
		max	77	80	720	52892

PC, pseudocyst; SCN, serous cystic neoplasm; IPMN, intraductal papillary mucinous neoplasm; MCN, mucinous cystic neoplasm; UNC, unclassified.

**Table 2:** Number of peptides detected in individual samples in the degradome dataset (<5 kDa aspirated fraction). Numbers of proteins from which these peptides originate are indicated in parentheses.

Cyst type	Peptides (proteins)	Peptides unique for group
CA	3224 (463)	660 (267)

SCN	2724 (353)	302 (128)

MCN	2175 (334)	117 (73)

IPMN	3072 (419)	226 (142)

PC	4730 (528)	982 (326)

Total	6996 (657)	2287 (494)

CA, carcinoma; SCN, serous cystic neoplasm; MCN, mucinous cystic neoplasm; IPMN, intraductal papillary mucinous neoplasm; PC, pseudocyst.

**Table 3:** Peptidases (P) and peptidase inhibitors (I) in the degradome fraction for which a different number of peptides were identified between combined CA, MCN, and SCN versus PC and IPMN groups.

UniProt ACC	Name	P/I	IPMN	PC	MCN	SCN	CA
P15085	Carboxypeptidase A1	P	53	64	6	7	19

P48052	Carboxypeptidase A2	P	11	14	0	1	3

P15086	Carboxypeptidase B	P	45	48	3	4	7

P08217	Chymotrypsin-like elastase family member 2A	P	7	7	1	1	2

P08218	Chymotrypsin-like elastase family member 2B	P	6	6	0	1	0

P09093	Chymotrypsin-like elastase family member 3A	P	9	8	3	1	3

P08861	Chymotrypsin-like elastase family member 3B	P	5	5	1	1	2

P07477	Trypsin-1	P	10	12	0	1	4

P07478	Trypsin-2	P	13	13	0	0	1

P00738	Haptoglobin	I/P	31	36	11	10	19

P04196	Histidine-rich glycoprotein	I	5	7	1	1	2

P01042	Kininogen-1	I	3	3	10	6	9

Q99895	Chymotrypsin-C	P	4	3	0	0	1

CA, carcinoma; SCN, serous cystic neoplasm; MCN, mucinous cystic neoplasm; IPMN, intraductal papillary mucinous neoplasm; PC, pseudocyst.

**Table 4:** Unique proteins for a given cyst type detected in proteome analysis.

**UniProt ACC**	**Name**	**Type of cyst**				
		**PC**	**MCN**	**SCN**	**CA**	**IPMN**
P08727	Keratin, type I cytoskeletal 19				**+**	

P07355	Annexin A2				**+**	

Q07654	Trefoil factor 3				**+**	

P04083	Annexin A1				**+**	

P55774	C-C motif chemokine 18			**+**		

P20142	Gastricsin		**+**			

P02549	Spectrin alpha chain, erythrocytic 1		**+**			

O43707	Alpha-actinin-4	**+**				

P05783	Keratin, type I cytoskeletal 18	**+**				

P35579	Myosin-9	**+**				

P15814	Immunoglobulin lambda-like polypeptide 1	**+**				

Q04118	Basic salivary proline-rich protein 3	**+**				

P08238	Heat shock protein HSP 90-beta	**+**				

Q06033	Inter-alpha-trypsin inhibitor heavy chain H3	**+**				

P67936	Tropomyosin alpha-4 chain	**+**				

Q06323	Proteasome activator complex subunit 1	**+**				

P27352	Gastric intrinsic factor	**+**				

P30046	D-dopachrome decarboxylase	**+**				

O43653	Prostate stem cell antigen	**+**				

CA, carcinoma; SCN, serous cystic neoplasm; MCN, mucinous cystic neoplasm; IPMN, intraductal papillary mucinous neoplasm; PC, pseudocyst.

## Data Availability

Mass spectrometry proteomics data have been deposited at the ProteomeXchange Consortium via the PRIDE [43] partner repository with the dataset identifiers PXD005248 and 10.6019/PXD005248.

## Supplementary Materials

Supplementary Materials
** Figure S1: coverage of serum albumin, fibrinogen alpha chain, apolipoprotein A-I, and alpha-1-antitrypsin in pancreatic cyst fluids.** CA, carcinoma; SCN, serous cystic neoplasm; MCN, mucinous cystic neoplasm; IPMN, intraductal papillary mucinous neoplasm; PC, pseudocyst.** Figure S2: dendrogram of significantly enriched (red) GO terms in the degradome dataset. Figure S3: dendrogram of significantly enriched (red) GO terms in the proteome dataset. Supplementary Tables ****1**-**13**** are deposited in separate excel file.**
