# Supplementary figures and images for: Mass Spectrometry-Based Comprehensive Analysis of Pancreatic Cyst Fluids
Source: Biomed Res Int. 2018 Nov 29;2018:7169595. doi: 10.1155/2018/7169595 (PMC6304507; doi:10.1155/2018/7169595)

Albumin

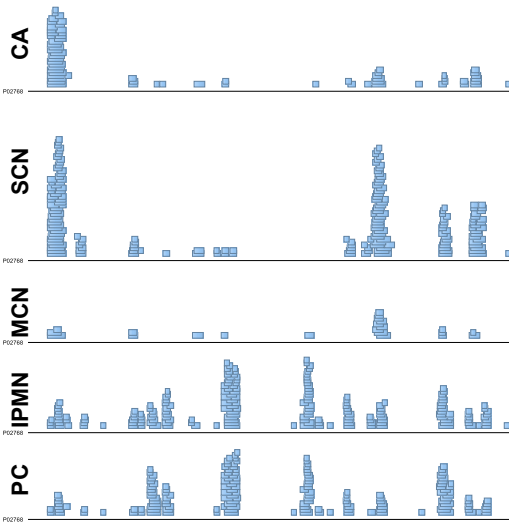

Fibrynogen alpha chain

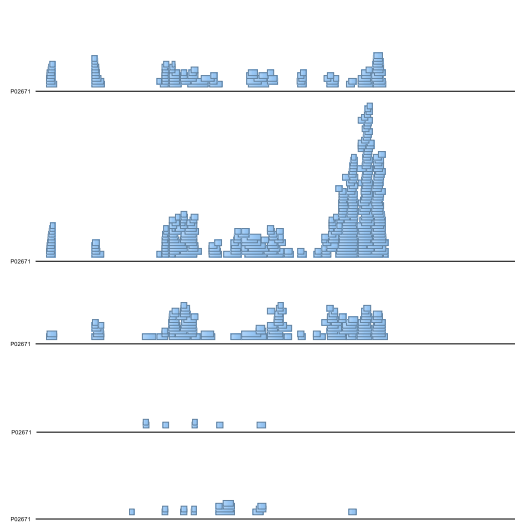

Alpha-1-antitrypsin

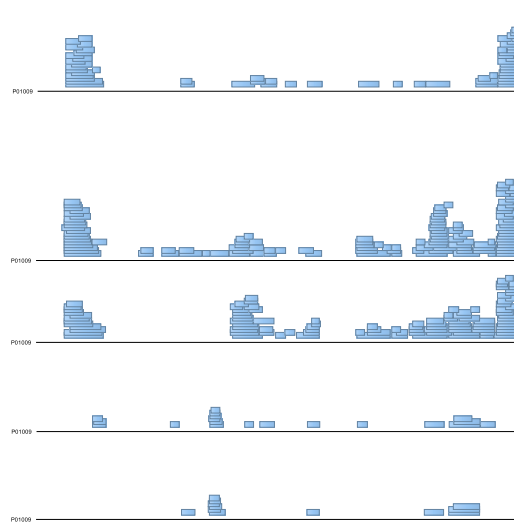

Apolipoprotein A-1

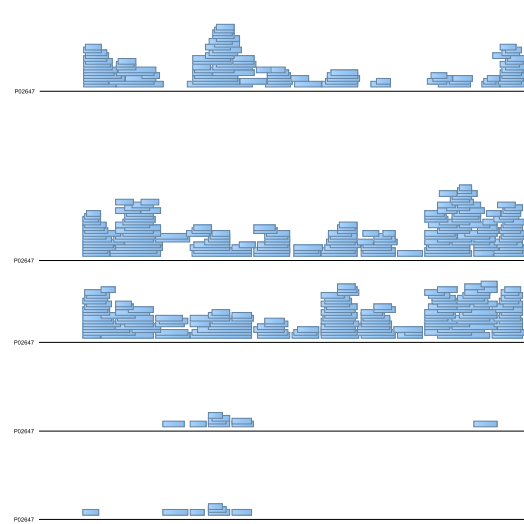

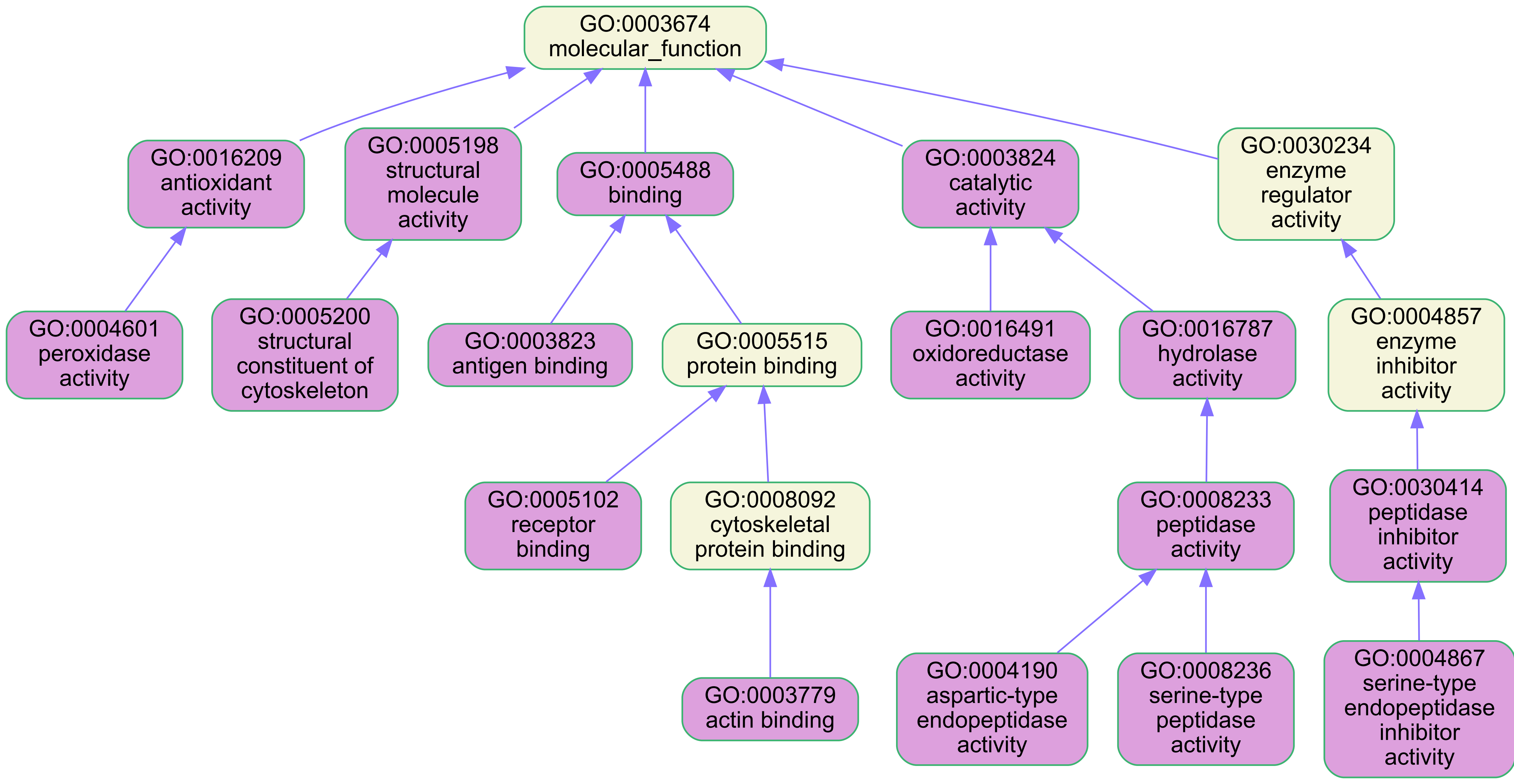

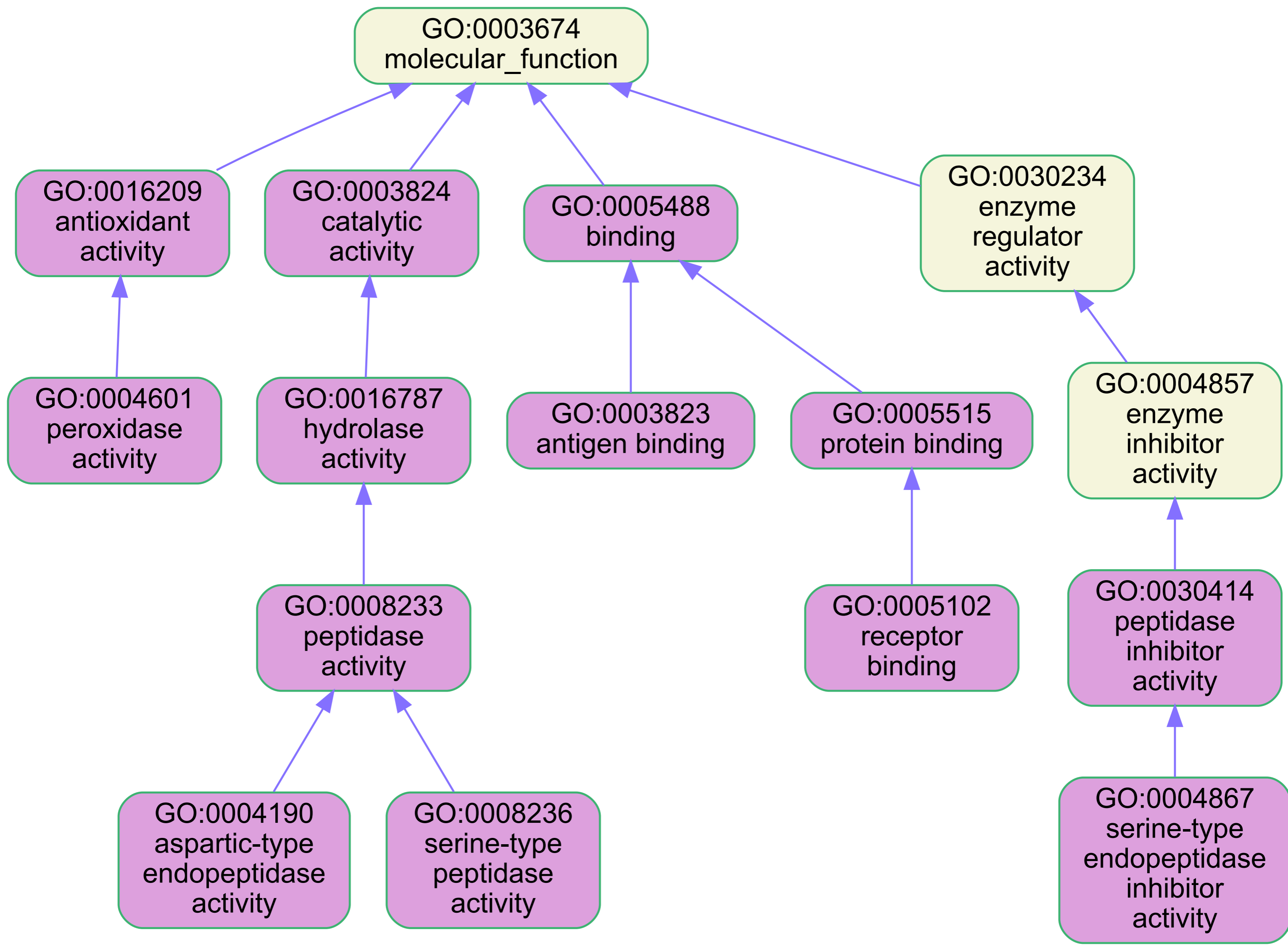

Supplement: Supplementary Materials — Figure S1: coverage of serum albumin, fibrinogen alpha chain, apolipoprotein A-I, and alpha-1-antitrypsin in pancreatic cyst fluids. CA, carcinoma; SCN, serous cystic neoplasm; MCN, mucinous cystic neoplasm; IPMN, intraductal papillary mucinous neoplasm; PC, pseudocyst. Figure S2: dendrogram of significantly enriched (red) GO terms in the degradome dataset. Figure S3: dendrogram of significantly enriched (red) GO terms in the proteome dataset. Supplementary Tables 1-13 are deposited in separate excel file. [file 7169595.f1.zip › Pages from 181109_Degradome_proteome_R2_clean_supplfig.pdf]
